# Joint Attention without Gaze Following: Human Infants and Their Parents Coordinate Visual Attention to Objects through Eye-Hand Coordination

**DOI:** 10.1371/journal.pone.0079659

**Published:** 2013-11-13

**Authors:** Chen Yu, Linda B. Smith

**Affiliations:** Department of Psychological and Brain Sciences, Cognitive Science Program, Indiana University Bloomington, Bloomington, Indiana, United States of America; Goldsmiths, University of London, United Kingdom

## Abstract

The coordination of visual attention among social partners is central to many components of human behavior and human development. Previous research has focused on one pathway to the coordination of looking behavior by social partners, gaze following. The extant evidence shows that even very young infants follow the direction of another's gaze but they do so only in highly constrained spatial contexts because gaze direction is not a spatially precise cue as to the visual target and not easily used in spatially complex social interactions. Our findings, derived from the moment-to-moment tracking of eye gaze of one-year-olds and their parents as they actively played with toys, provide evidence for an alternative pathway, through the coordination of hands and eyes in goal-directed action. In goal-directed actions, the hands and eyes of the actor are tightly coordinated both temporally and spatially, and thus, in contexts including manual engagement with objects, hand movements and eye movements provide redundant information about where the eyes are looking. Our findings show that one-year-olds rarely look to the parent's face and eyes in these contexts but rather infants and parents coordinate looking behavior without gaze following by attending to objects held by the self or the social partner. This pathway, through eye-hand coupling, leads to coordinated joint switches in visual attention and to an overall high rate of looking at the same object at the same time, and may be the dominant pathway through which physically active toddlers align their looking behavior with a social partner.

## Introduction

People look to where their social partners look. The coordination of visual attention among social partners is central to many components of human behavior, including joint action[1], [2] and spoken communication [3]–[5].The ability to coordinate visual attention with a social partner appears especially important to early language learning [6], [7] and may be the key limiting factor in both language learning and social development for children with autism [8]. Contemporary research [6]–[13]concentrates on eye-gaze following as the essential mechanism through which visual attention is socially coordinated. Here we present evidence from one-year-olds and their parents for an alternate pathway, through the coordination of hands and eyes in goal-directed action.

The evidence for developmentally early eye-gaze following is unambiguous: Newborns make faster saccades to the onset of a peripheral target when cued by a lateral eye movement in the same direction[12], [13]. However, this early sensitivity is evident only given a stationary frontal view of the face and visual targets that are just to the right or left of midline [13], [14]. Other evidence indicates that eye-gaze direction in and of itself is not easily used by infants, or even adults, in more complex contexts such as when the view of the face deviates even slightly from a frontal view and especially when head and eye direction are discordant[6], [14]–[18]. Further, when there are more than two potential targets that are spatially near or on the same side of midline, two-year-olds completely fail to follow the single cue of gaze direction, even given a still and frontal view of the face[16]; even older children and adults also have considerable difficulty in this context [19]–[21]. In brief, early gaze following by human infants is a reliable phenomenon but it is limited to simple spatial contexts. Furthermore, gaze following by infants was demonstrated in discrete experimental trials with clear and repeated signals from caregivers, that enhanced the temporal availability of gaze signals. These limitations in the spatial acuity and temporal stability of gaze direction indicate a serious gap in contemporary understanding of how infants and their mature caretakers coordinate visual attention in the complex social contexts of everyday life with moving heads and eyes, different and changing views of faces, and multiple not-well-separated visual targets. One possibility is that the proficiencies seen in infants' abilities to follow the looking behavior of others in the laboratory do not scale up to these real-world contexts.

Here, we pursue a different possibility, that there is an alternative pathway to the dynamic coupling of parents' and infants' visual fixations in social interactions. This possibility was suggested by recent observations using head-mounted cameras placed on older infants as they physically engaged in various activities. None of these studies, conducted by several different research groups and for different purposes, was directly concerned with joint attention; nonetheless, the researchers all noted the lack of faces in the head-camera images even in contexts in which the infant was socially engaged with an adult[22]–[26]. Although faces were infrequent in the infant's head camera views, hands –the infant's own and the hands of their social partners– were commonly in the head-camera images. Hands that act on objects provide an alternate and spatially precise route to coordinated visual attention: When reaching for an object, actors (both infants and adults) systematically shift their eye gaze to a reaching target just before they begin to move their hand and continue looking to the object as the hand travels to that location[24], [27]–[29]. The direction, timing and velocity profiles of hand and eye movements in the service of goal-directed action are strongly correlated (e.g., 27–31). Thus, eye gaze is a faster cue to the hand's target object but the hand trajectory is a temporally extended cue that is more spatially precise (at contact) and more stable compared with gaze. Further, during object manipulation, people direct their gaze almost exclusively toward target objects to gather visual information for guiding movements [28], [30], [31]. Thus, because hand and eye movements provide redundant information about the target of a goal-directed action, one could –in contexts that included the handling of objects –follow a social partner's *visual* attention without looking at the eyes at all, that is, by following the partner's hand actions. Because hand actions are both spatially precise and temporally stable, this alternate route to socially coordinated visual attention should be robust and stable even in dynamically and spatially complex tasks in which there are moving heads, multiple shifts in attention, and multiple and moving targets with unrestricted locations.

**Figure 1:**
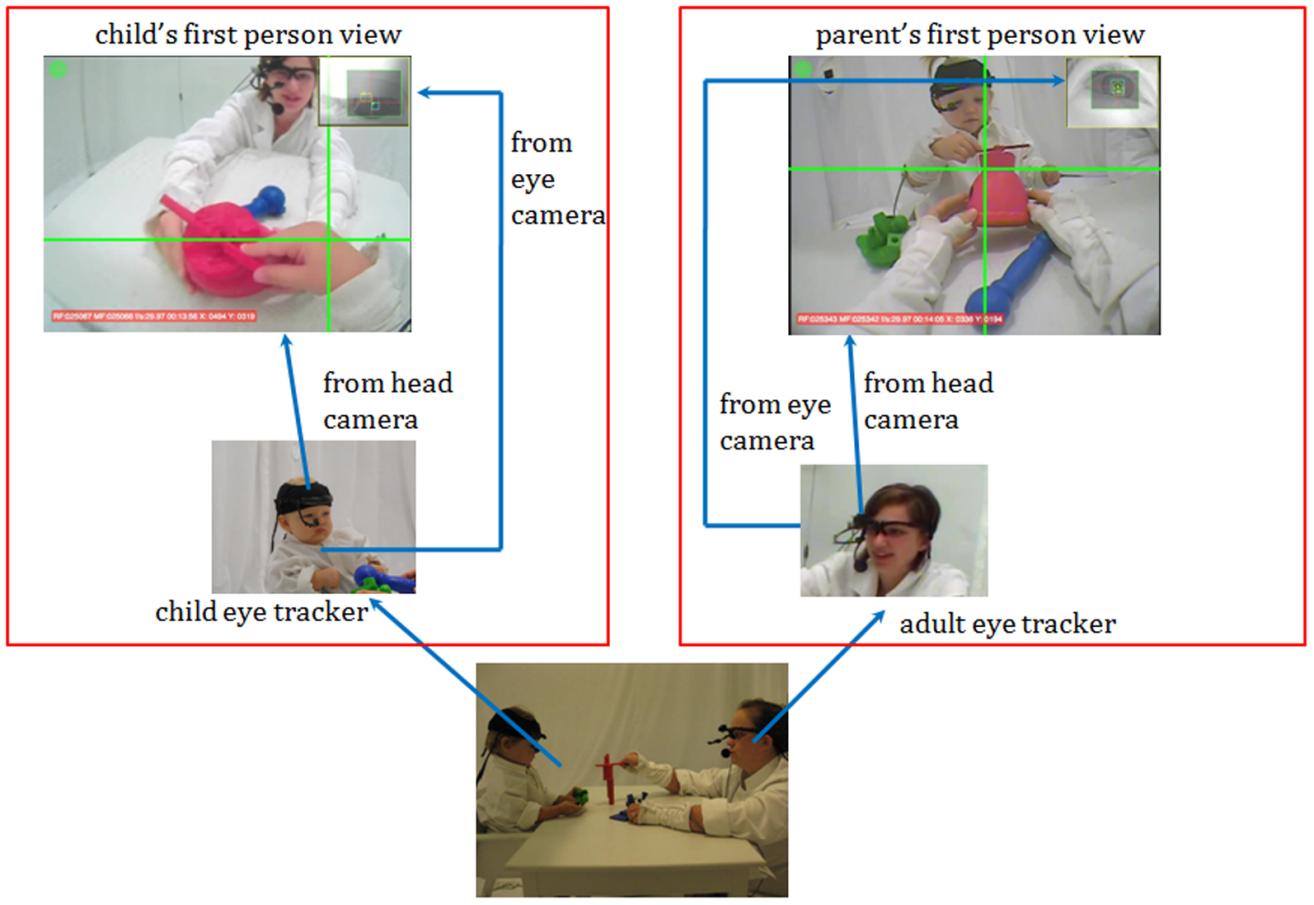
A dual eye tracking experimental paradigm. Infants and parents played with a set of toys on a tabletop in a free-flowing way. Both participants wore a head-mounted eye tracker that recorded their moment-to-moment gaze direction from their egocentric views. The subject of the photograph has given written informed consent, as outlined in the PLOS consent form, to publication of their photograph.

To assess this possibility, we asked parents and their one-year-olds to wear head-mounted eye tracking systems (see Video S1)that captured both the head-centered scene and the participants' fixations in that scene as parents and infants played together with a set of novel toys (3 objects at time, shown in Figure 1). There were no constraints or instructions on how to engage with the toys, other than to play as normally as possible (see Video S3). By tracking the momentary visual fixations of each participant, we could measure how often they attended to the same object at the same time, the specific cues used to coordinate visual attention, the temporal lags in following a partner's shift in visual attention, and the potentially different roles of parents and infants.

## Results

**Figure 2:**
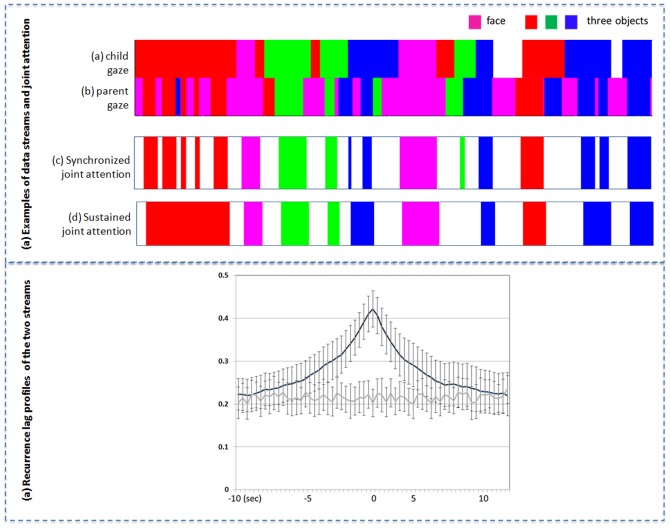
Gaze data and joint attention measures. (a) Examples of a coupled ROI stream from one infant (first row) and parent (second row), with each color indicating a different object or the social partner's face. Coordinated attention is measured as synchronized joint attention (third row) and sustained joint attention (fourth row, see text for definition). (b) Mean recurrence lag profiles. Cross recurrence of parent-child gaze data at different time lags is compared with a randomized baseline.

The 3toy objects on the table and the partner's face were defined as regions-of-interest (ROIs) for frame-by-frame measures of parents' and infants' eye fixations. Figure 2(a) shows a representative example of the raw fixation streams for one dyad and Table 1 provides the summary statistics for several measures for the entire sample. Infants showed more sustained fixations on ROIs than did parents, consistent with previous measures of their often long durations of looking to a single target [32]. Infants looked at their parent's face rarely, consistent with prior head-camera studies[23], [24], whereas parents were fixated on their infant's face over a third of the frames. Overall, parents visually monitored all the objects and as well as the child's face with rapid switching among these regions of interest whereas the infant's system was slower and consisted of longer looks and only rare looks to the parent's face.

**Table 1:** Measures of visual attention.

	Infant	Parent	Infant-Parent comparison
Fixations to ROIs			
Frequency (rate/min)	30.74	56.16	t(16) = 13.90
	(5.41)	(10.65)	p<.001, d = 3.04
Duration (ms)	2068	946	t(16) = 8.79
	(642)	(322)	p<.001, d = 2.24
Looks to face			
Proportion of time	11.63	37.21	t(16) = 7.15
	(7.81)	(12.66)	p<.001, d = 2.49
Frequency (rate/min)	4.56	20.92	t(16) = 14.54
	(2.01)	(4.45)	p<.001, d = 5.14
Duration (ms)	1557	989	t(16) = 3.35
	(363)	(492)	p<.004, d = 1.31
Looks to objects			
Proportion of time	62.13	47.92	t(16) = 5.53
	(5.78)	(10.66)	p<.001, d = 1.66
Frequency (rate/min)	16.88	31.98	t(16) = 11.04
	(4.20)	(7.18)	p<.001, d = 2.57
Duration (ms)	2334	948	t(50) = 9.80
	(982)	(318)	p<.001, d = 1.90

Means and standard deviations (in parentheses) of properties of infant's and parent's visual attention during joint play. **Frequency** measures the number of looks per minute; **Proportion of time** measures the overall proportion of time that participants looked at ROIs; and **Duration** captures the average duration of looks.

Coordinated vision between parents and infants was defined as looks to the same ROI(same object or each other's face) and a lag-based cross-recurrence between the parent's and the infant's ROI streams was calculated for each dyad[4], [33]. Figure 2(b) shows the mean recurrence lag profile, with 0 indicating simultaneous fixations to the corresponding ROI and the curve to the left indicating the looks to an ROI by the infant that were followed by the parent looking to the same ROI within the defined lag, and the pattern to the right indicates parent's looks to an ROI that were followed by a look to the same ROI by the infant within the defined lag. The symmetry of the profile indicates that infants and parents both led and followed their partner's attention as equal participants. The cross-lag baseline was generated by randomly pairing infants and parents, with controls for the overall individual dynamic properties of infants and parents as the source of the observed pattern. A 2 (real vs. randomly paired) ×61 (lag times) mixed effects analysis of variance (treating a recurrence profile as a distribution of temporal data, and lag as a repeated measure factor) revealed a significant main effect (F(1, 120) = 1072.03, p<0.001, η^2^ = 0.856). The sharp peak in coordinated looking around 0 ms and the higher recurrence relative to baseline within a5 sec lag in each direction implicates strongly coordinated looking behavior between infants and parents.

From these recurrence measures, we defined *Synchronized* and *Sustained* measures of joint attention as illustrated in Figure 2. The synchronized measure is the simultaneous (frame-by-frame) co-occurrence of parent and infant fixation on the same ROI. The sustained measure was defined by joining successive moments of synchronized attention on the same object that were separated by less than 300 ms of looks elsewhere and each combined segment was required to be at least 500 ms long (see Materials and Methods for details). Table 2 summarizes a set of statistics on these two measures, both of which indicate a high degree of coordination especially for looks to objects: Parents and infants looked at the same toy object at the same time .33 and .42 of the time by the synchronous and sustained measures respectively; however, they looked at each other's faces at the same time only 0.10 and .09 of the time by these two measures respectively. The high overall degree of coordinated attention to objects obtained even though participants switched their fixations among ROIs, achieving on average over 23 distinct bouts of joint attention per minute by the simultaneous measure and over 9 per minute by the sustained measure. Looks to the partner's face by the infant were not frequent (4.56 per minute, shown in Table 1) and thus seem an unlikely basis for this tight coupling of looking behavior to objects.

**Table 2:** Overall patterns of coordinated attention.

		synchronized attention	sustained coordinated attention
**Proportion (% of time)**	Overall	42.56 (11.35)	51.35(5.41)
	mutual gaze	9.76 (5.85)	9.45(3.23)
	Object	32.80 (7.63)	41.55(6.46)
**Frequency (rate/min)**	overall	22.58(5.06)	9.29(1.63)
	mutual gaze	4.85(2.37)	2.24(1.38)
	object	17.73(4.19)	7.05(1.63)
**mean duration (in second)**	overall	0.85 (0.30)	2.45(0.95)
	mutual gaze	0.86(0.28)	1.85(0.41)
	object	0.82(0.31)	2.53(1.02)

1) **proportion of joint attention** measures the overall proportion of time that participants were in coordinated attention by the two definitions. 2) **frequency of joint episodes** measures the number of coordinated attention events per minute; and 3) **mean duration** captures the average duration of joint attention events (of synchronization without even momentary looks away, or the duration of longer sustained bouts of attention with potential looks away by a partner within that bout). For each measure, the results are further divided into an **overall** measure (counting all the joint attention moments including both shared attention on objects and on each other's face) and then the two components of that overall measure, shared attention to **objects** (counting only the moments of jointly attending the same object) and **mutual gaze** (counting only the moments of looking at each other's face).

During the play sessions, parents and infants actively handled and acted on the objects. One or both of the participants were holding an object during .93 of the time (SD = .08). Neither parent nor infant hand actions on objects dominated the interaction, the infant was handling an object on average .25 of the time (SD = .14), the parent was handling an object .25 of the time (SD = .16), and they were both handling an object or objects at the same time .43 of the time (SD = .22). These active hand actions provide a potential route to coordination visual attention.

**Figure 3:**
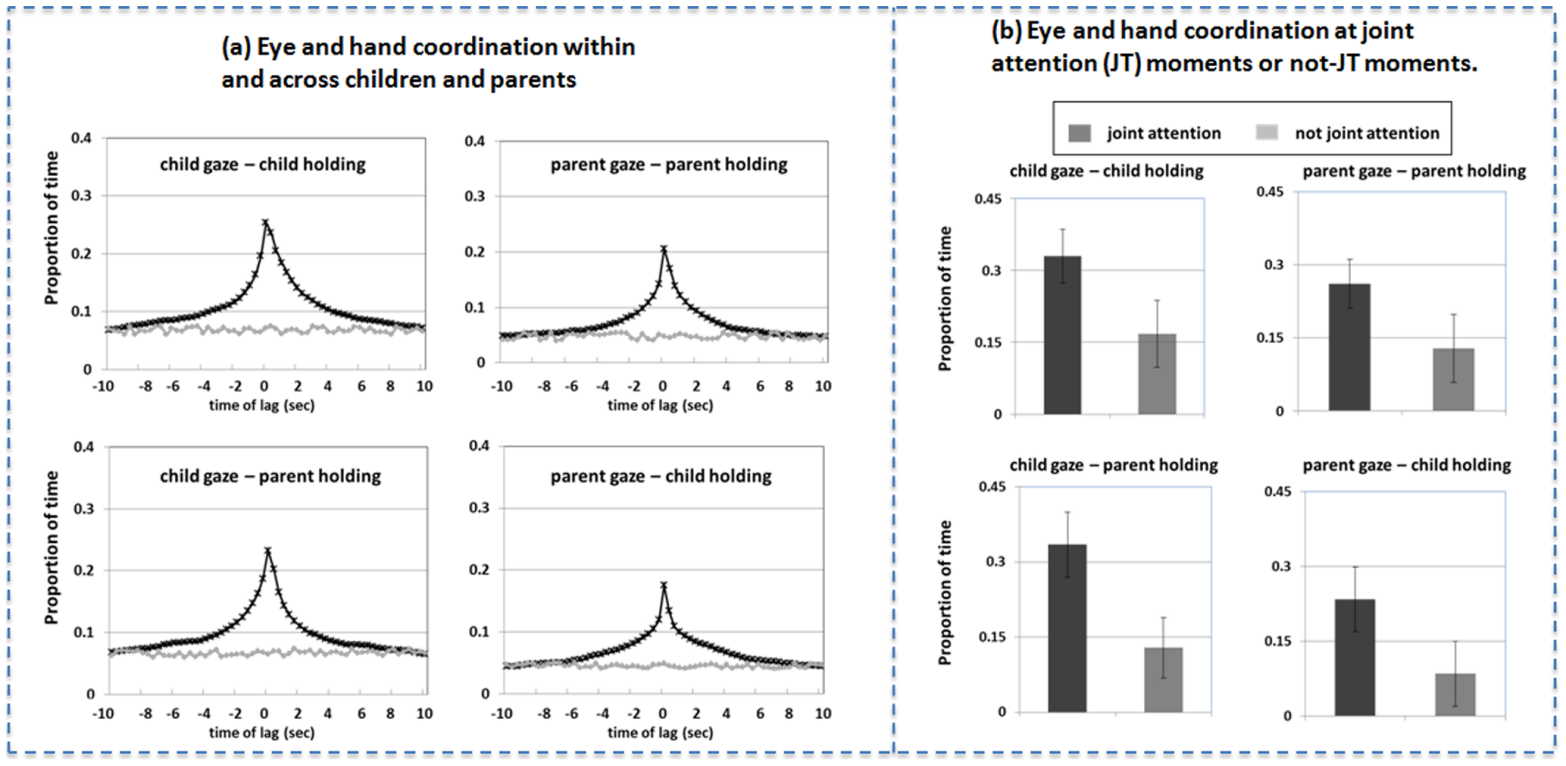
Eye-Hand coordination in parent-child interaction. (a) Recurrence lag profiles between eyes and hands within each participant (top two panels) and across two social partners (bottom two panels) show close couplings within and across two individuals in the whole interaction. (b) Eye-Hand coordination within each participant (top two panels) and across two social partners (bottom two panels) at joint attention moments and moments without joint attention. Eyes and hands are more closely coupled at joint attention moments.

Figure 3(a) shows evidence with respect to the components of that coordination. One component is the redundancy of hand and eye direction within an individual, measured by the *within-individual* (child or parent) recurrence of the actor's eye fixation to the actor's own hand action on an object. A second component is visual sensitivity to the social partner's hand actions, measured by the *across-individual* (child gaze with parent hands, parent gaze with child hands) recurrence of the observer's eye fixation to the actor's hand action on an object. Compared to baselines generated by randomly pairing parents and children, there is strong eye-hand coordination within and across partners around time lag 0 with eyes often fixating on the object being touched by the individual or by the partner. The eye-hand coordination *within* the child was tighter than *within* the parent (F(1, 120) = 298.76, p<0.001, η^2^ = 0.706) and the *across* partner coordination of child-eye and parent-hand was tighter than the coordination between parent-eye and child-hand (F(1, 120) = 405.61, p<0.001, η^2^ = 0.760). Overall then, the infants' looking behavior was more tied to hand activities by both the infant and the parent.

Figure 3(b) shows the cross recurrence measures at time lag 0 for the four types of eye-hand coordination within and across individuals. Coordination was tighter at joint attention moments than at moments without joint attention (eye-hand within child: t(16) = 4.778; eye-hand within parent: t(16) = 3.513; child's eyes and parent's hands: t(16) = 6.243; parent's eyes and child's hands: t(16) = 7.025; in all of the cases, p<0.001, d>1.266). In sum, because the actors' hands and eyes are tightly coupled, the non-acting partner may coordinate looking with the acting partner by looking to where the actor's hands are. For all of these measures, the link between holding behavior and fixation on an object at joint attention moments was stronger for infants than parents (within individuals: t(16) = 3.702, p<0.005, d = 1.265; across individuals: t(16) = 4.510, p<0.001, d = 1.302), indicating a stronger dependency on manual behaviors –by self and partner – in organizing the one-year- olds' looking behavior.

The symmetrical cross-recurrence plots of parent-infant fixations in Figure 2 (b) show that both parents and infants were equally likely to initiate a look that was quickly followed by the other partner. The frequency with which parents and infants handled objects also indicates that they were partners in the interaction and that parents were not controlling the activity by showing objects nor were they merely following their infants' interests. Further, if we use sustained joint attention episodes as the measure, the infant led (and the parent followed) on 46.2% of the episodes (and thus the parent led and the infant followed on 53.8% the episodes, t(16) = 0.61; p = .48), a result that again indicates the coordination of two active and equal participants. Moreover, infants and parents appear equally fast in following each other's lead. In the cases of child leading (parent following), it took parents, on average, 1023 ms (SD = 465 ms), to join the child in attending to an object; it took the child 825 ms (SD = 321 ms) from the onset of the parent's first look to the onset of the child's look to that same object (mixed-model analysis using lmer, β = 0.67; p = 0.14). In sum, the overall pattern shows that one year olds and their parents are equal partners with both manually engaged with the objects and both leading and rapidly following shifts in their partner's focus of visual attention.

**Figure 4:**
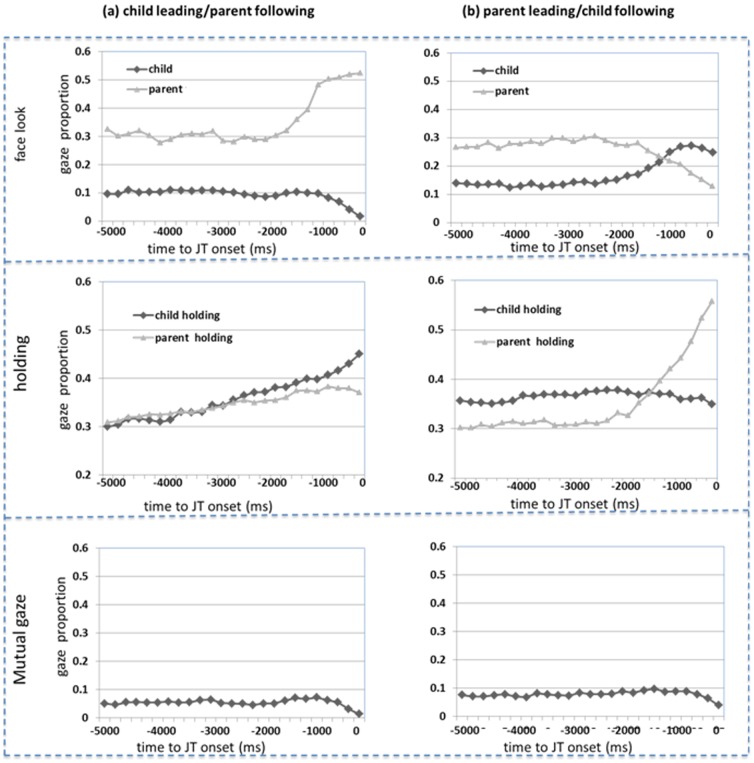
Dynamic patterns of behaviors before joint attention. Dynamic patterns of three behaviors from the 5 second before the onset of coordinated attention, in either child-leading (left column) and parent-leading (right column) cases. **Top**: the proportion of time that either child or parent looked at each other's faces prior to joint attention. Only in the case of child leading, an increase of face look from parent to the child's face started around 2000 ms before the onset of joint attention. **Middle**: the proportion of time that either child or parent was holding the to-be-jointly-attended object prior to joint attention. In the case of child leading, both children and parents increasingly held the target object. In the case of parent leading, only the probability that the parent held the target object was dramatically increased before joint attention. **Bottom**: the proportion of time that they looked at each other's faces in both child-leading and parent-leading cases shows little change of mutual gaze.

We next calculated the temporal profiles of three types of behaviors – face look, object holding and mutual gaze – that could precede a sustained joint attention bout and did so separately for bouts in which the child was the first to look to the object (and the parent followed) versus when the parent first looked to the object (and the child followed).We first determined the frame in which a sustained joint attention bout began and then determined (frame by frame) whether the child and/or the parent was looking at the other's face during the 5 seconds preceding the onset of joint attention (see [34], for use of this approach in time-course analyses). Figure 4 (top two panels) shows how often (on average) a follower looked to the leader's face prior to a sustained joint attention episode. The figure also shows how often the leader looked back to the follower's face, perhaps to check on whether their attentional lead was being followed. Although parents and infants were equal partners in leading and following, when they followed the other partner's attention, they differed in their use of face and hand information. The parent's looked to infant face as well as hands, suggesting a role for eye-gaze following. There is no evidence, however, that the infrequent looks of infants to their parent's faces played any role in guiding the infant's looks to the objects. More specifically, when parents followed their infants' look to an object (top left in Figure 4), they systematically looked to the child's face more, starting at about 2000 ms prior to the onset of coordinated attention. Within a temporal window of 2 seconds prior to the onset (60 samples within the window), a comparison with the overall face-look baseline (0.372) shows a significant increase of looking at the child's face by the parent (t(59) = 4.086, p<0.001, d = 1.414), reaching a peak of 0.535 before the onset. When infants followed a parent's look to an object, they also looked reliably more to the parent's face compared with the baseline 0.116 (t(59) = 3.846, p<0.001, d = 1.192). However, the peak of the infants' looks to the parent's face was only 0.273 before the onset of coordinated attention and thus these looks, at best, can explain only a small proportion of the observed coordinated attention episodes through gaze following. In sum, gaze following by the parent seems a likely –but not sole – contributor to the parent's following their child's attentional interest; gaze following by the infant seems unlikely to have played a significant role in the infant's following of the parent's attention.

Consistent with the proposed pathway through eye-hand coordination both within and across participants, object holding by both partners was associated with joint attention. Figure 4 (middle row) shows holding activities by both children and parents before joint attention in child leading (left) and parent leading (right) cases. Joint attention bouts often began with one partner holding and looking at a target, and then the other partner looking at the held object. When parents led, within a 2-second window before the onset of joint attention bouts, the leading parent was more likely to hold the attended object relative to baseline (0.305) of holding one of three objects (t(59) = 5.303, p<0.001, d = 3.030), indicating that the following infant used holding actions signaled by the parents to join the parent's attentional lead. Also when parents led, there was no significant difference of infants' holding the target object relative to baseline (t(59) = 1.784, p = 0.105).However, when infants led, both parents and children were more likely to hold the attended object before joint attention bouts (t_parent_(59) = 3.481, p_parent_<0.001, d_parent_ = 1.923; t_child_(59) = 5.303, p_child_<0.001, d_child_ = 3.030). Again, toddlers were more dependent than their parents on manual behaviors of their partner to follow their partner's attentional lead. Further, the partner who initiated a sustained joint attention bout was more likely to hold the object than was the other partner. When parents led, they were more likely to hold the attended object (t(59) = 3.551, p<0.002, d = 0.925) and when infants led, they were more likely to hold the attended object (t(59) = 9.682, p<0.001, d = 3.881).

Finally, parents and infants did simultaneously gaze into each other's face by both the synchronized and sustained measures (Table 2). However, these moments of mutual gaze show no relation to the onset of sustained joint attention bouts as shown by the probability profiles of mutual gaze in Figure 4 (bottom row). As is apparent, instantaneous mutual gaze appears to have little dynamic relation to joint attention. The pattern offers no obvious role for sustained mutual gaze in organizing bouts of joint attention in both child-leading (t(59) = 1.89, p = 0.074) and parent-leading (t(59) = 1.866, p = 0.068) cases. The lack of relation between mutual gaze and joint attention does not imply that moments of mutual gaze are not developmentally meaningful, as they may play an important role in emotional regulation and social development in other ways than through joint attention [35], [36].

## Discussion

**Figure 5:**
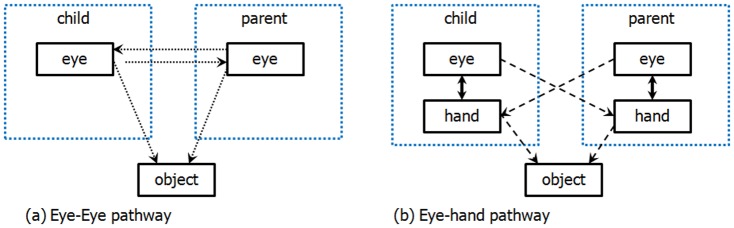
Multiple pathways lead to joint attention. (a) Joint attention is achieved through following the other's gaze direction. (b) Joint attention is achieved through hand following (dash lines) pathways because eye-hand coordination within an agent (solid lines) ensures the same object either through eye direction and hand activities.

One-year-olds and their parents temporally coordinated their visual attention to objects and did so smoothly, consistently, and as equal partners without one partner dominating or leading the interaction. Further, the two partners often shifted attention to the same objects together in time. Figure 5(a) illustrates one pathway to coordinated visual attention: each partner looks to the other's eyes and the seen direction of gaze of the partner influences the direction of the other partner's gaze, leading to coupled looking behavior. Figure 5(b) illustrates an alternate pathway: Within individuals a tight coordination of hand and eye in goal-directed action means that hand and eye actions present spatially redundant signals but with the hand cue being more spatially precise and temporally stable. The results show that the hand actions of an actor have a direct effect on the partner's looking, leading to coordinated *visual* attention without direct gaze following. This hand-eye pathway is used by one-year-olds and their parents, and supports a dynamic coordination of the partners' fixations that is characterized by rapid socially coordinated adjustments of looking behavior. The documentation of a functional alternative to following the eye gaze of a social partner begins to fill the contemporary knowledge gap in understanding just how joint attention between infants and parents might work in cluttered and complex everyday contexts [17]–[21]. Joint attention as a means of establishing common reference is essential to infant learning in many domains including language [37] and the present results show how coordinated looking may be established and maintained in spatially and dynamically complex contexts that include manual actions on objects. Infant attention and sensitivity to hand actions demonstrated in the present results is also consistent with the large and growing literature on their ability to interpret the causal implications of hand movements and gestures [38].

Successful adult social interactions are known to depend on rapid (with fractions of a second) behavioral adjustments in response to and across a suite of sensory-motor behaviors that include eye, head, hand, mouth, and posture movements [39]–[41]. The hand-eye pathway evident in one-year-olds and their parents shows this same character of well-coordinated rapid adjustment in response to the partner. In contexts in which the social partners are manually engaged with objects, toddlers may not often look to the face and eyes of parents because there is a more precise, more readable and more reliable cue to the parent's momentary intentions in the interaction. How frequent are these contexts in everyday life, when objects are not just looked at but also touched by the social partners? We know of no direct evidence on this question; certainly there are times when infants and parents interact by jointly looking (and pointing) to distant objects. But the daily lives of toddlers – mealtime, toy play, getting dressed – are filled with social contexts in which objects are handled. The tendency to watch hand actions –over eye-gaze shifts – is most likely greater in some contexts than in others, such as the present one with novel and interesting toys, or in the contexts of instrumental acts such as making sandwiches or feeding a doll. Over time the tight association of hand and eye gaze direction in these kinds of contexts may support the development of finer spatial discriminations of eye gaze as well as the integration of eye, hand, and posture cues, a conjecture that is consistent with a recent computational model showing that learning to recognize gaze direction can be achieved through hand detection [42].Active physical engagement with objects seems to be an ideal training ground for learning about subtler predictive bodily cues to attention and intention because –once hand contact is made –there is no uncertainty as to the intended target. Current approaches [43]that concentrate on looks to faces and eyes, and to teaching those looks as parts of intervention programs for individuals with various developmental delays may be making the task harder than the more natural multi-cue developmental route. Although the present findings focused on hands as potent cues to a social partner's visual attention, the developing and mature system is likely to include multiple kinds of cues including vocal cues as well as body movements. In the present context of one-year-olds and novel objects with no known names, it is unlikely that parents provided lexical cues that could specify the momentary object of their attention; however, prosodic cues, perhaps rhythmically coupled to their hand actions, could well play a role in cueing and entraining joint shifts in attention [44]. In brief, the present finding point to the importance of casting the net larger than eye-gaze direction, if we want to understand the developmental origins and mechanisms underlying joint attention.

West and King [45] proposed that development could be considered as a series of ontogenetic niches that present individuals with an ordered set of different tasks under different constraints. Human newborns are motorically altricial and are often placed or held to stabilize the head. The relative immobility of very young infants (and their heads) may constrain the spatial structure of social interactions, as the caretaker puts her own face near and directly in front of the infant and also bring objects in hands close to the infant that are centered or to one side of midline. In this context, an early sensitivity to eye-gaze direction limited to still frontal facial views and a potential target to one side or the other of midline may be sufficient to couple the visual attention of parents and infants, and begin an initial tuning and integration of bodily cues. It is seductive when one sees a precocious glimmer of adult competency, such as the effects of lateral gaze shifts on newborns' looking in spatially constrained contexts, to generalize the phenomenon to other contexts at other developmental stages, and assume that the key component for the adult competency has been found. As infants reach, then sit, then crawl, then walk, they travel through a series of very different developmental niches that may recruit and strengthen different pathways to successful social interactions. Although gaze following is clearly an important mechanism supporting human social interactions and one whose developmental role begins early, the robust flexibility that characterizes skilled human social interactions across a variety of social tasks may depend not on a single route, but on the development of a network of overlapping and partially redundant pathways with multiple degenerate routes to the same end.

## Materials and Methods

### Ethics Statement

All research was approved by the Research Subjects Review Board at the Indiana University (protocol #05-10318). Parents volunteering their infants for the study were fully informed of the study procedures and completed written informed consent and permission forms in advance of the study.

### Participants

Infants within 3 months of their first birthday were recruited. The final sample consisted of 17 parent-infant dyads. Mean age of participating infants was 13.5 mo (range 11 to 15 mo); 8 additional infants began the study but refused to wear the measuring equipment.

### Stimuli

There were 6 unique novel “toys” that were constructed from wood and plastic in the laboratory with examples shown in Figure 1. The 6 unique toys were organized into two sets of three so that each object in the set had a unique uniform color. Each novel toy was a complex object made from multiple and moveable parts. They were all of comparable overall size, on average, 288 cm^3^.

### Experimental setup

Parents and infants sat across from each other at a small table (61 cm×91 cm×64 cm). Parents sat on the floor such that their eyes and heads were at approximately the same distance from the tabletop as those of the infants, a posture that parents reported to be natural and comfortable. Both participants wore head-mounted eye trackers (positive science, LLC; see Video 1). Each eye-tracking system included an infrared camera – mounted on the head and pointed to the right eye of the participant – that records eye images, and a scene camera (see in Figure 1) capturing the first-person view from the participant's perspective. The scene camera's visual field was 90 degrees, providing a broad view but one less than the full visual field of toddlers (approximately 180°,). Each eye tracking system recorded both the egocentric-view video and gaze direction (x and y) in that view, with a sampling rate of 30 Hz. Another high-resolution camera (recording at 30 frames per sec) was mounted above the table and provided a bird's eye view that was independent of participants' movements.

### Procedure

Three experimenters worked together to test each parent-infant dyad. Upon entering the experiment room, the infant was seated in the chair and a push-button pop-up toy was placed on the table. One experimenter played with the infant while the second experimenter placed the eye-tracking gear low on the forehead of the infant at a moment when the child was engaged with the toy (see Video S1). To collect calibration points for eye tracking, the first experimenter then directed the infant's attention toward an attractive toy positioned on the table while the second experimenter recorded the timing, that is, the moment, at which the infant looked at the object (see Video S2). This procedure was repeated 15 times with the toy placed in various locations on the tabletop to ensure a sufficient number of calibration points that were used after the session to calibrate eye-gaze within the head camera images. To calibrate the parent's eye tracker, the experimenter asked the parent to look at one of the objects on the table and repeated the procedure at different locations to obtain 15 calibration points from the parent.

Parents were told that the goal of the experiments was to study how parents and infants interacted with objects during play and therefore they were asked to engage their infants with the toys and to do so as naturally as possible. Each of the two sets of toys was played with once for 2 min at time, resulting in 4 minutes of free-play data from each dyad. Order of sets was counterbalanced across dyads.

### Data Annotation

#### Gaze Data

Four regions-of-interest (ROIs) in each play trial were defined: three toy objects and the partner's face. These ROIs were coded manually by a coder who watched the first-person view video with a cross-hair indicating gaze direction, frame-by-frame, and annotated when the cross-hairs overlapped any portion of an object or face and which ROI. Thus, each dyad provided two gaze data streams containing four ROIs as shown in Figure 2. The second coder independently coded a randomly selected 10% of the frames with 95% agreement.

#### Hand action

Holding behaviors (who and which object) from infants and parents were coded manually, frame-by-frame, from the images captured by the overhead camera. The second coder also independently coded a randomly selected 10% of the frames with 96% agreement.

### Cross-Recurrence measures of two gaze data streams

**Figure 6:**
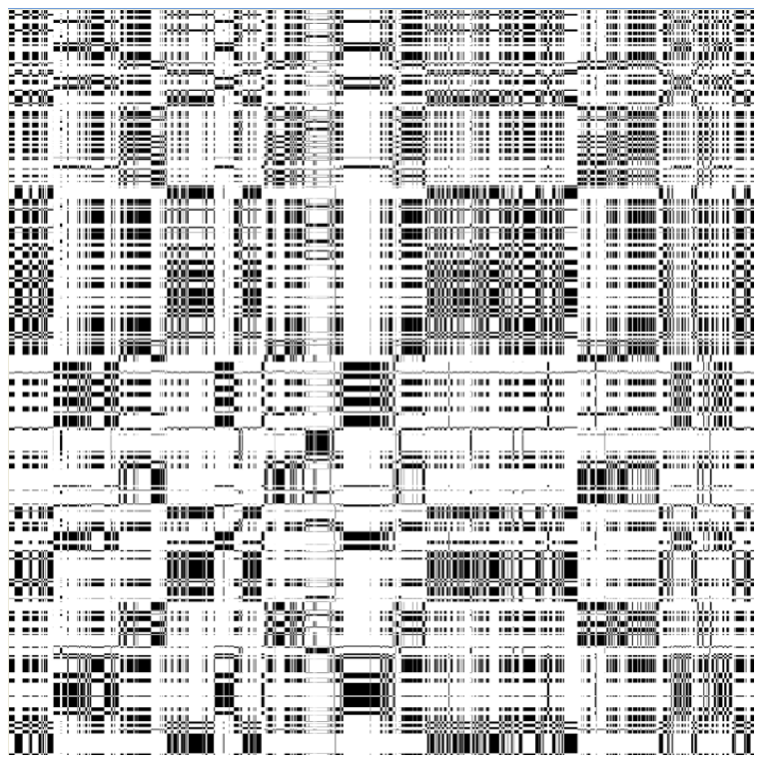
An example recurrent plot from a pair of participants. Eye movement recurrence at 0(block boxes) along the diagonal line indicate that two participants not only generated overall joint attention moments but also dynamically switched their attention together from one object to the other as time goes by.

We used cross-recurrent measures for quantifying both coordinated attention between infants and parents, and eye-hand coordination within and across two individuals. In all the cases, we aligned two categorical temporal data streams (e.g. ROIs of which objects were gazed at or which objects were held), and measured their temporal coupling with different degrees of lags. Here we use cross-recurrence measures of two gaze data streams as an example to explain the method, and the same method was also applied to measure the coupling between gazing and holding data. In the plot shown in Figure 6, the horizontal dimension represents the child gaze stream over time and the vertical dimension represents the parent gaze data over time. The central diagonal provides a measure of synchrony in looking to the same object with the black pixels on the diagonal indicating the child and the parent were fixating within the same ROI at the same time, and the white pixels indicating that the child and the parent were not gazing at the same ROI. Other pixels in the non-diagonal areas of the plot reflect time-shifted recurrences (that is, correspondences) between the child's and the parent's gaze, a measure of whether parent's and infant's look to the same object but with some delay (time unit: 1/30 second) as one partner follows the other to that object. The pixels below the diagonal correspond to child leading and the pixels above indicate parent leading. As shown in Figure 1, overall, the plot for this dyad demonstrates coupled attention and well-coordinated gaze shifts as indicated by the sequence of black blocks along the diagonal. To quantify the patterns of coordination in these two data streams, we generated cross-recurrence lag profiles with the following steps. We first computed the percentage match (or “cross-recurring”) along the diagonal line in a cross-recurrence plot, which reflects synchronization between two data streams without any delay in time. We then took parallel diagonals along the primary diagonal line and derived a percentage match for each parallel diagonal – each match reflects how much “cross-recurring” took place at a time lag. By computing this percentage match across all lags, we generated a *diagonal-wise recurrence lag profile* reflecting the pattern of coordination between the two data streams at different degrees of lag, as shown in Figure 2(b) and Figure 3(a) in the main text.

### Measures of sustain joint attention

In an effort to quantify coordinated attention between infants and parents, we operationally defined two measures: 1) Synchronized joint attention; 2) Sustained joint attention. Moments of synchronized joint attention was measured by aligning the two parent-infant ROI data streams and computing whether two social partners concurrently attended to the same ROI frame by frame. The proportions were calculated by counting all the moments in the interaction including those moments that eye trackers might not record participants' gaze direction due to technical difficulties (roughly 8% from parents, and 15% from infants); and those moments that either parent or child might look at somewhere else (off the joint play task) but not the partner's face or three objects (7% from parents, and 12% from infants). These were included as looking elsewhere and therefore not as joint attention moments which means the reported joint attention moments are likely underestimates.

The analyses of the dynamics of parent's and infant's eye movements show spontaneous but brief gaze shifts between the social partner's faces and other objects during periods of otherwise extended attention to the same object (e.g. looking at object A for a long while with briefly interleaved looks to the other's face or other objects). Accordingly, the measure of sustained joint attention was defined to capture joint fixations on one object that were sustained – dominating for a long period of time. This measure of sustained joint attention was calculated by joining successive moments of synchronized attention using the following rules: segments of synchronized attention by parent and toddler to the same object were considered part of a single bout of joint attention if they were temporally separated only by brief looks elsewhere (shorter than 300 ms, etc.). As a result, this operation merges small joint attention segments sharing the same object into a single longer bout. Next, those merged bouts based on synchronized attention were filtered by excluding those that were less than 500 ms. Thus, only merged bouts greater than 500 ms were considered to be sustained joint attention. Compared with synchronized joint attention, those two additional operations led to fewer but long episodes as sustained joint attention as shown in Figure 2.

## Supporting Information

Video S1 Experimenters put a head-mounted eye tracker on an infant's forehead and adjusted the angles of the eye and scene camera at the moments when the child was engaged with some toys.
(MOV)

Video S2 Experimenters calibrated the head-mounted eye tracker by asking an infant to look various locations on the tabletop.
(MOV)

Video S3 An example of two synchronized first-person view videos captured by two head-mounted eye trackers on both infant's and parent's forehead. The cross-hair in a video indicates gaze direction in the first person view.
(MOV)
